# The Application of Lophatherum Gracile Brongn Flavonoids in Wheat Flour Products: Effects on the Structural and Functional Characteristics of Wheat Dough

**DOI:** 10.3390/foods13162556

**Published:** 2024-08-16

**Authors:** Qin Li, Yi Liu, Huimei Bao, Haihua Zhang

**Affiliations:** 1 School of Food Science and Technology, Jiangsu Food and Pharmaceutical Science College, No. 4 Meicheng Road, Huaian 223003, China; bhm7412@126.com; 2 College of Food and Health, Zhejiang Agriculture and Forestry University, No. 666 Wusu Road, Linan District, Hangzhou 311300, China; liuyi@zafu.edu.cn

**Keywords:** lophatherum gracile brongn flavonoid, wheat dough, multiscale structure, water distribution

## Abstract

The effects of lophatherum gracile brongn flavonoids on the multiscale structure and functional properties of wheat dough were investigated. Wheat dough samples with varying contents of lophatherum gracile brongn flavonoids were analyzed to assess changes in thermal-mechanical rheological properties, microstructure, chemical interactions, water distribution, and macropolymer formation by Mixolab mixer, fluorescence microscopy, and low-field nuclear magnetic resonance (LF-NMR). The findings revealed that lophatherum gracile brongn flavonoids disrupted the three-dimensional network of gluten proteins in the wheat dough, leading to decreased water-binding capacity and reduced gluten protein crosslinking while enhancing thermal stability and inhibiting the starch retrogradation of the dough. This study provided important insights into the interaction mechanisms between lophatherum gracile brongn flavonoids and the proteins/starch in wheat dough, offering theoretical guidance for the development of novel wheat-based products for industrialization and practical production.

## 1. Introduction

As a widely consumed food globally, wheat-based products play a crucial role in the development of staple foods, with their quality, texture, and nutritional value significantly influencing the evolution of the culinary landscape [1,2]. Wheat dough, serving as the cornerstone of these products, exerts a decisive impact on the quality of wheat-based foods due to its structural and functional attributes. Wheat dough’s mechanical properties, nutritional composition, and technological characteristics are crucial factors that determine the final quality of products in the food industry. Hence, investigating the structure and functional properties of dough holds paramount importance for both theoretical understanding and practical applications in the realm of food science and technology.

In the food industry, the application of natural food additives is increasingly gaining attention [3]. Among them, lophatherum gracile brongn flavonoids, as a type of natural plant extract with various physiological activities such as antioxidant, antimicrobial, and anticancer properties, have been widely utilized in the functional food field [4,5,6,7]. Lophatherum gracile brongn, belonging to the poaceae family, is traditionally used both as food and medicine; it is primarily found in the Zhejiang and Jiangsu provinces of China, growing in mountainous areas, forests, or shaded areas below 1200 m above sea level [8]. Flavonoids are important functional substances found in lophatherum gracile brongn and have been industrially produced [7]. Flavonoids constitute a complex mixture, including flavones, flavonols, and phenolic acid compounds. The main components of lophatherum gracile brongn flavonoids include orientin, homoorientin, vitexin, and isovitexin [6].

Flavonoids, a type of polyphenol, have been demonstrated to influence the structure and rheology of dough [9,10,11]. Research indicates that flavonoids present in Tremella fuciformis powder and barley flour can affect the protein composition and secondary structure of dough [12]. While specific investigations regarding the impact of pure flavonoid compounds on the gluten network structure in model dough systems are limited, the presence of flavonoids in ingredients such as hulless barley is associated with alterations in the rheological properties of dough [13,14]. Additionally, the effects of adding the flavonoid dihydroquercetin on enzymatic processes during dough ripening and the antioxidant properties of the final bread product have been studied [15].

Although flavonoid compounds have been shown to affect the physicochemical and structural properties of dough, there is still a lack of in-depth research on the impact of lophatherum gracile brongn flavonoids on the structure and functional properties of wheat dough. Therefore, this study aimed to investigate the effects of lophatherum gracile brongn flavonoids on the structure and functional properties of wheat dough. Analytical tools such as the Mixolab mixer, fluorescence microscopy, and low-field nuclear magnetic resonance (LF-NMR) were employed to analyze the impact of adding lophatherum gracile brongn flavonoids at different concentrations on the thermal-mechanical rheological properties, microstructure, chemical interactions, water distribution, and macropolymer changes of the dough. The study aimed to elucidate the mechanism of interaction between lophatherum gracile brongn flavonoids and proteins and starch in wheat dough, providing theoretical guidance for the development of novel wheat-based products.

## 2. Materials and Methods

### 2.1. Materials

Medium-gluten wheat flour (Jinlongyu brand, 11.3% protein content, 24.6% wet gluten content) was purchased from Baolong Supermarket (Hangzhou, China). Lophatherum gracile brongn flavonoids (flavonoid content ≥90%, with an orientin and isoorientin content of 10.03%) were obtained from Zhejiang Shengshi Biotechnology Co., Ltd. (Hangzhou, China). Lauryl sodium sulfate (SDS), bovine serum albumin (BSA), dithiothreitol (DTT), 5,5′-dithio-bis-(2-nitrobenzoic acid) (DTNB), trimethylolaminomethane (Tris), glycine, trichloroacetic acid (TCA) and all the reagents and chemicals were of analytical grade and purchased from Shanghai Macklin Biochemical Co., Ltd. (Shanghai, China).

### 2.2. Preparation of Samples

To obtain dough samples with lophatherum gracile brongn flavonoid contents of 0%, 0.3%, 0.5%, and 0.8% (*w*/*w*), a precise amount of wheat flour was accurately weighed out. Different masses of lophatherum gracile brongn flavonoids were added according to volume ratios to achieve a final mass of 200 g. After adding an equal mass of distilled water, the mixture samples were thoroughly blended in a food processor. Dough containing lophatherum gracile brongn flavonoids was prepared following the predetermined maximum water absorption and constant 5-min rule as per the AACC (2010) International Approved Method 54-60.01.

### 2.3. Determination of the Microscopic Morphology of the Dough Samples

The method for determining the microscopic morphology of the dough was a modification of the method described by Wang [16]. The freshly prepared dough samples (1.5 cm in length and 0.5 cm in diameter) were frozen at −80 °C overnight. The samples were transferred to a specimen holder to achieve a final height of approximately 7 mm, secured with glue, and immediately refrozen overnight. After freezing, the samples were sectioned into 30 μm slices and stained for 1 min with a mixture of 0.1 mg/mL Rhodamine B and 0.25% fluorescein isothiocyanate (FITC). The stained samples were then observed, and images were captured using a laser scanning confocal microscope (LSM 710, German Zeiss Company, Oberkochen, Germany).

The imaging parameters were as follows: Objective magnification 40×, eyepiece magnification 10×, FITC excitation wavelength 488 nm, emission wavelength 528 nm, Rhodamine B excitation wavelength 543 nm, emission wavelength 626 nm. Five images were taken for each sample (215 × 215 μm size, 1024 × 1024 pixels).

### 2.4. Determination of the Thermal-Mechanical Properties of the Dough Samples

The method for determining the thermal-mechanical properties of the dough was a modification of the method described by Huang [17]. The rheological properties of the dough samples were determined using the Chopin + testing program on a Mixolab (French Chopin Tech Company, Paris, France). The experimental parameters were set as follows: dough hook speed of 80 r/min, dough mass of 75 g, dough consistency stabilized at 1.10 ± 0.05 N·m, water tank temperature maintained at 30 °C, and a moisture basis of 14%. The temperature program for the experiment included an initial isothermal period at 30 °C for 8 min, followed by a temperature increase to 90 °C at a rate of 4 °C/min and held for 7 min, and finally a temperature decrease to 50 °C at a rate of 4 °C/min and held for 5 min; the entire process lasted 45 min.

### 2.5. Determination of the Chemical Interactions in the Dough Samples

Chemical interactions in the dough were measured according to the modified method by Cao [18]. Firstly, the following reagents were prepared using 0.05 M pH 7.0 phosphate buffer: (1) 0.05 M NaCl (PA); (2) 0.6 M NaCl (PB); (3) 0.6 M NaCl + 1.5 M urea (PC); (4) 0.6 M NaCl + 8 M urea (PD); (5) 0.6 M NaCl + 8 M urea + 50 mM DTT (PE).

Subsequently, the freshly prepared dough samples were freeze-dried, ground, and sieved through a 177 μm mesh screen. Then, 200 mg of sample powder was accurately weighed and dissolved in 5 mL of each reagent, shaken at room temperature for 120 min, and centrifuged at 4 °C and 10,000 rpm for 15 min. 0.1 mL of the supernatant was taken, added to 0.9 mL of water, and then mixed with 5 mL Coomassie Brilliant Blue G-250, followed by vortex mixing and standing at room temperature for 5 min. The absorbance was measured at 595 nm using a UV-visible spectrophotometer (UV-1800, Inesa Analytical Instrument Co., Ltd., Shanghai, China). Finally, a standard curve was prepared using bovine serum albumin as a reference to calculate the protein content in each solution, where ionic bonds = PB-PA, hydrogen bonds = PC-PB, hydrophobic interactions = PD-PC, and covalent bonds = PE-PD.

### 2.6. Determination of the Glutenin Macropolymer Content of the Dough Samples

The method for determining the GMP content in the dough was a modification of the method described by Han and Wang [16,19]. Firstly, the freshly prepared dough samples were freeze-dried, ground, and sieved through a 177 μm mesh screen. 0.5 g of sample powder was accurately weighed into a 20 mL solution of 1.5% sodium dodecyl sulfate (SDS) and shaken at 25 °C for 60 min. Subsequently, the mixture was centrifuged at 10,000 rpm for 15 min, and the supernatant was discarded. The above procedure was repeated once, and the final precipitate was retained, and its nitrogen content was determined using the Kjeldahl method to quantify the GMP content.

### 2.7. Determination of the Water Distribution in the Dough Samples

The method was a modification of the method described by Assifaoui [20]. The transverse relaxation time (T2) of the dough samples was determined using a low-field 1 H nuclear magnetic resonance (NMR) analyzer (NMI20-040V-I, Niumag Co., Ltd., Suzhou, China). A 2 g dough sample was sealed with a layer of plastic film and placed in an NMR glass tube with a diameter of 25 mm. T2 were tested at 32 °C using the Carr-Purcell-Meiboom-Gill (CPMG) pulse sequence. The relaxation curve was fitted with an exponential equation. Following the classification by Hu (2022), T2 was divided into three categories: T21 (0.1–10 ms), T22 (10–100 ms), and T23 (>100 ms), corresponding to bound, entrapped, and free water, respectively. T21 (0.1–10 ms) was further categorized into 0.1–1 ms and 1–10 ms, representing vicinal water and multilayer water, respectively.

### 2.8. Statistical Analysis

The gathered data were expressed as the average value ± standard deviation of at least triplicate determinations. Significance was differentiated using a one-way analysis of variance (ANOVA) followed by Tukey’s test at the level of *p* < 0.05 using Statistical Package for the Social Sciences (SPSS) 20.0 software (SPSS Incorporated, Chicago, IL, USA). The figures were plotted using Origin 2022 software (OriginLab Corporation, Northampton, MA, USA).

## 3. Results and Discussion

### 3.1. Effect of Lophatherum Gracile Brongn Flavonoids on the Microstructure of the Dough

**Figure 1:**
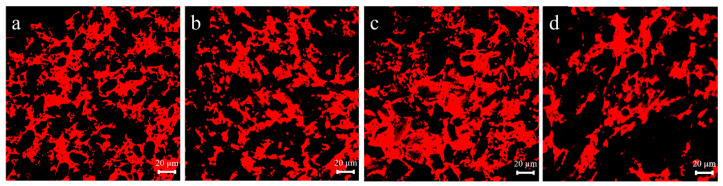
The microstructure of dough samples with different bamboo leaf flavonoid contents. (**a**–**d**) represented dough samples with lophatherum gracile brongn flavonoid contents of 0%, 0.3%, 0.5%, and 0.8%, respectively.

The microstructure of the dough gluten network with different amounts of lophatherum gracile brongn flavonoids added is shown in Figure 1. From the figure, the gluten network of the dough can be clearly seen. The gluten network structure of the dough samples without added lophatherum gracile brongn flavonoids was uniformly dense, with the best uniformity in the three-dimensional gluten network structure, the best continuity of gluten and protein nodes, and minimal porosity (Figure 1a). When the amount of added lophatherum gracile brongn flavonoids increased from 0.3 g/100 g to 0.8 g/100 g, the gluten network structure of the dough samples gradually became looser, and fracture points and large voids appeared. When the addition reached 0.8 g/100 g, the porosity of the gluten network was at its maximum, and the continuity of the gluten fibers was poor (Figure 1d). This indicated that the addition of lophatherum gracile brongn flavonoids disrupted the three-dimensional gluten network structure of the dough, resulting in uneven continuity of the gluten network structure, and this disruptive effect increased gradually with the increasing amount of added lophatherum gracile brongn flavonoids. Li’s study added Ginkgo biloba powder rich in flavonoids to dough and observed a reduction in the stability of starch and gluten network interactions [21]. Similarly, Yu’s research also reported consistent findings [22].

### 3.2. Effect of Lophatherum Gracile Brongn Flavonoids on the Thermal-Mechanical Properties of the Dough

The Mixolab mixer was used to test the thermal-mechanical properties of dough samples in order to further verify the impact of lophatherum gracile brongn flavonoids on the starch molecules and gluten proteins in the dough [23]. The rheological behavior of the dough during heating and cooling cycles, simulating the process from raw to cooked dough, was assessed by the Mixolab [24]. During the constant temperature stage, the characteristics of water absorption rate and gluten strength during dough formation were tested; during the heating, high-temperature, and cooling stages, the weakening of gluten, starch gelatinization, and retrogradation characteristics were tested [24]. C3–C2 represented the gelatinization characteristics of the starch, with a higher value indicating stronger gelatinization properties [25]. C3–C4 represented the thermal stability, with a lower value indicating greater thermal stability [26,27]. C5–C4 represented the retrogradation characteristics of the starch, with a lower value indicating less tendency for retrogradation [28,29].

**Figure 2:**
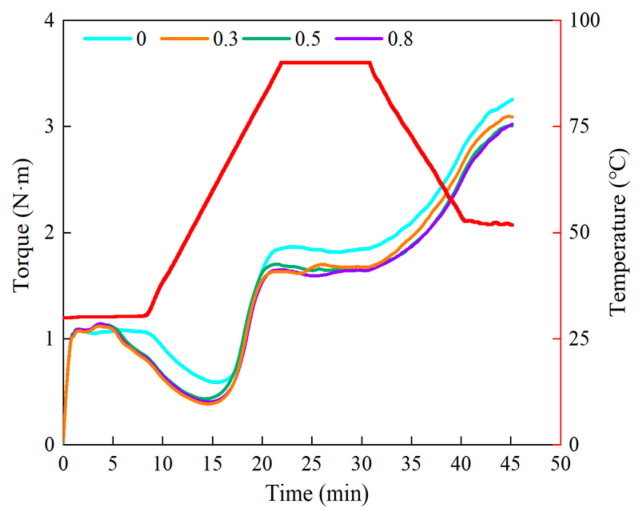
The thermal-mechanical properties of dough samples with different bamboo leaf flavonoid contents.

From Figure 2, it can be observed that dough samples with different contents of lophatherum gracile brongn flavonoids underwent significant changes after the heating and cooling cycles. Specifically, with an increase in the content of lophatherum gracile brongn flavonoids, water absorption, formation time, and stability time gradually decreased (Table 1), indicating that the addition of lophatherum gracile brongn flavonoids disrupted the gluten network structure and intermolecular interactions. Additionally, this may be due to the competition for water between lophatherum gracile brongn flavonoids and gluten or starch, leading to a reduction in cross-linking between gluten proteins, thereby reducing dough stability and formation time. The decrease in C3–C2 with increasing lophatherum gracile brongn flavonoid content suggested that lophatherum gracile brongn flavonoids disrupted the gelatinization characteristics of the dough, resulting in samples with greater resistance to expansion during the heating process. The decrease in C3–C4 with increasing lophatherum gracile brongn flavonoid content indicated that lophatherum gracile brongn flavonoids enhanced the thermal stability of the dough. The decrease in C5–C4 with increasing lophatherum gracile brongn flavonoid content suggested that the addition of lophatherum gracile brongn flavonoids hindered the absorption of water by starch in the dough, thereby inhibiting the gelatinization rate and retrogradation rate of the starch. These results indicated that lophatherum gracile brongn flavonoids could disrupt the gluten network structure and compete for water with the gluten or starch, making the dough more resistant to expansion during the heating gelatinization process, thus having a negative impact on starch gelatinization but enhancing the thermal stability of the dough. This was also because the lophatherum gracile brongn flavonoids hindered the absorption of water by starch in the dough, thereby suppressing the retrogradation of the starch.

### 3.3. Effect of Lophatherum Gracile Brongn Flavonoids on the Chemical Interaction Properties of the Dough

**Figure 3:**
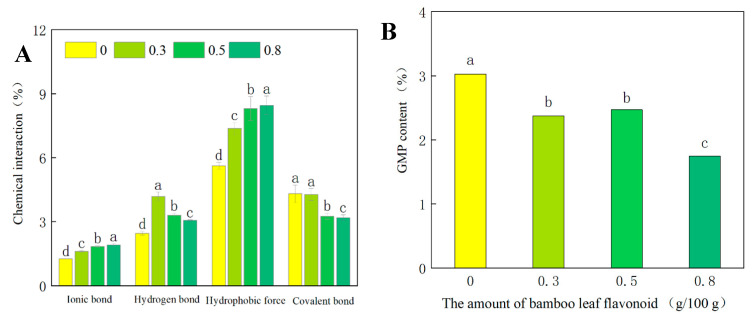
Effect of bamboo leaf flavonoids (BLF) on the chemical bonds (**A**) and the glutenin macropolymer content of the dough (**B**). (Letters a to d represent a significant difference between the data (*p* < 0.05)).

Different types of chemical bonds play crucial roles in the formation and properties of dough gluten networks. Therefore, by investigating the different types of chemical bonds in dough samples, a better understanding of the influence of lophatherum gracile brongn flavonoids on the structure and properties of dough can be achieved. The trends in ionic bonds, hydrogen bonds, hydrophobic interactions, and covalent bonds with varying lophatherum gracile brongn flavonoid contents are summarized in Figure 3A. It can be observed from the figure that as the content of lophatherum gracile brongn flavonoids increased from 0 to 0.8 (g/100 g), the ionic bonds increased from 1.27 to 1.92, and hydrophobic interactions increased from 5.62 to 8.45. This suggested that lophatherum gracile brongn flavonoids mainly interacted with gluten proteins and starch through ionic bonds and hydrophobic interactions, supporting the structure of the dough in the form of non-covalent complexes. Covalent bonds decreased with the increase in lophatherum gracile brongn flavonoid content, with a less significant decrease when the content of lophatherum gracile brongn flavonoids was 0.3. This may be because low levels of lophatherum gracile brongn flavonoids were insufficient to break the covalent bonds of gluten proteins, and a more noticeable decrease in covalent bonds occurred when the content of lophatherum gracile brongn flavonoids increased to a certain amount. After the addition of lophatherum gracile brongn flavonoids, the hydrogen bond content of the dough decreased with increasing addition, but overall remained higher than in the blank control group (without added lophatherum gracile brongn flavonoids). This may be because when the content of lophatherum gracile brongn flavonoids was low, the role of hydrogen bonds was more prominent. As the content of lophatherum gracile brongn flavonoids increased, the roles of ionic bonds and hydrophobic interactions became dominant in the dough, weakening the role of hydrogen bonds. The hydroxyl groups of lophatherum gracile brongn flavonoids could form hydrogen bonds with the side chains of the starch and the carboxyl groups of gluten proteins [16], resulting in a higher total number of hydrogen bonds compared to the blank control group. Some studies have suggested that interactions between proteins and flavonoids could occur via both covalent and non-covalent bonds, which contribute to the structure, formation, and development of dough [30,31].

### 3.4. Effect of Lophatherum Gracile Brongn Flavonoids on the Glutenin Macropolymer Content of the Dough

The glutenin macropolymer (GMP) is a large molecular aggregate formed by the intra- and intermolecular S-S bonds between low molecular weight glutenin subunits (LMW-GS) and high molecular weight glutenin subunits (HMW-GS) in wheat gluten [32,33]. Its content reflects the distribution of glutenin polymer particles and can serve as a predictive indicator for the gluten strength and quality of dough-based products. The GMP content in dough samples with different contents of lophatherum gracile brongn flavonoids is shown in Figure 3B. With increasing lophatherum gracile brongn flavonoid content, the GMP content showed an overall decreasing trend. When the lophatherum gracile brongn flavonoid content reached 0.8 (g/100 g), the GMP content decreased to 1.75%. This suggested that the addition of lophatherum gracile brongn flavonoids inhibited the formation of glutenin protein crosslinking aggregates based on disulfide bonds in the dough, resulting in a reduction in gluten strength and a gradual loosening of the gluten network structure. This result was consistent with the findings of microstructure and chemical interaction analyses. Zhang studied the impact of tannic acid addition on dough structure, revealing that tannic acid, due to its reducing properties, disrupted disulfide bonds in wheat dough [34].

### 3.5. Effect of Lophatherum Gracile Brongn Flavonoid on Water Distribution in the Dough

The interactions between water, proteins, and starch in dough significantly influence the distribution and flow of water, playing a crucial role in the physicochemical properties of dough and the structure of gluten networks [35]. The transverse relaxation time variations of the dough samples with different contents of lophatherum gracile brongn flavonoids were determined using LF-NMR. Among them, T21 represents bound water, which can tightly bind with macromolecules such as gluten proteins and starch, exhibiting poor mobility [36]. T21 (0.1–10 ms) is further divided into 0.1–1 ms and 1–10 ms, corresponding to vicinal water and multilayer water, respectively [37]. T22 represents entrapped water, which is trapped within the starch and gluten protein network, exhibiting intermediate mobility between bound water and free water [36,38]. T23 represents water in the dough that can freely flow between the components [38]. A shorter T2 relaxation time indicates a lower mobility of water molecules, suggesting a tighter binding between the water and the components.

**Figure 4:**
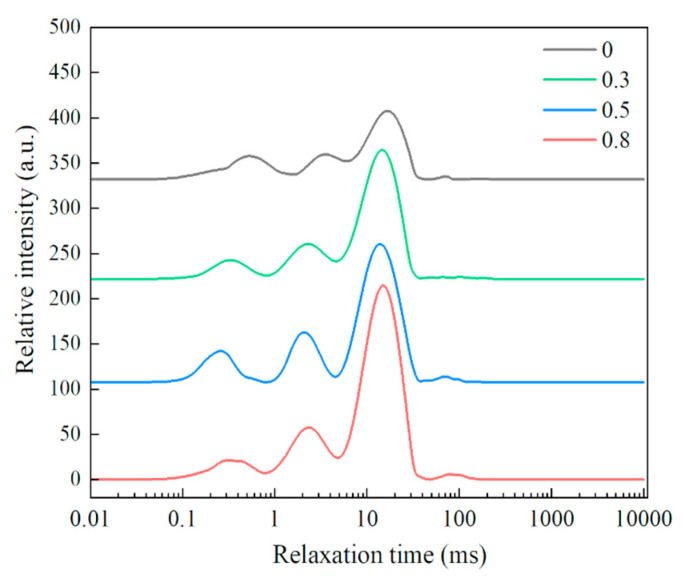
Water distribution of dough samples with different bamboo leaf flavonoid contents.

From Figure 4 and Table 2, it can be observed that with an increase in lophatherum gracile brongn flavonoid content, there was a gradual reduction in vicinal water, while multilayer water, entrapped water, and free water increased progressively. This indicated that lophatherum gracile brongn flavonoids led to a decrease in the water-binding capacity of the dough and reduced the cross-linking of gluten proteins, causing some of the water that was tightly bound to proteins or starch particles to migrate out of the gluten protein network and transform into entrapped water and free water. Combining this with the results of the microstructure and chemical interaction properties mentioned above, it was evident that with the increasing addition of lophatherum gracile brongn flavonoids, the gluten network structure of the gluten proteins gradually became loose, with ionic bonds and hydrophobic interactions playing the predominant roles in the dough. This facilitated the mobility of water molecules within the dough.

## 4. Conclusions

Phenolic compounds act as antioxidants by scavenging free radicals, thus potentially preventing chronic diseases. Studies have shown that incorporating phenol-rich substances in dough-based products significantly enhances their antioxidant capacity [15]. Methods to improve the bioavailability and utilization of phenolic compounds in dough products include incorporating phenol-rich fractions like bran, adding promising natural phenolic substances or extracts, and using enzymes or mechanical strength to release bound phenolic compounds. Lophatherum gracile Brongn, a phenolic compound derived from natural plant extracts, possesses various physiological activities such as antioxidant, antimicrobial, and anticancer properties, and has been widely utilized in functional food applications [4,5,6,7]. However, research on the impact of lophatherum gracile Brongn on wheat dough remains scarce. This study aimed to investigate the feasibility of incorporating bamboo leaf flavonoids into wheat dough and explore their effects on dough structure and functional properties.

The present study demonstrated that lophatherum gracile brongn flavonoids disrupted the three-dimensional network structure of gluten proteins and the interactions among components in wheat dough, with this disruptive effect increasing gradually with increasing flavonoid content. The addition of lophatherum gracile brongn flavonoids disrupted the covalent bonds, leading to their primarily ionic and hydrophobic interactions with proteins and starch in the wheat dough. This resulted in uneven continuity of the gluten network structure, a decrease in the water-binding capacity of the wheat dough, and a reduction in gluten protein crosslinking. However, it enhanced the thermal stability of the wheat dough and inhibited starch retrogradation.

## Figures and Tables

**Table 1:** The thermal-mechanical properties of dough samples with different bamboo leaf flavonoid contents.

BLF Amount (g/100 g Wheat Flour)	Water Absorption (%)	Formation Time (min)	Stable Time (min)	C3–C2 (Nm)	C3–C4 (Nm)	C5–C4 (Nm)
0	60.70 ± 0.00 a	5.70 ± 0.03 a	9.12 ± 0.14 a	1.29 ± 0.01 a	0.07 ± 0.01 a	1.50 ± 0.01 a
0.3	60.50 ± 0.00 b	4.40 ± 0.05 b	5.45 ± 0.05 b	1.27 ± 0.01 a	0.08 ± 0.00 a	1.46 ± 0.02 b
0.5	60.40 ± 0.00 b	3.64 ± 0.10 c	4.95 ± 0.04 c	1.25 ± 0.01 b	0.09 ± 0.00 a	1.43 ± 0.01 b
0.8	60.10 ± 0.00 c	3.57 ± 0.08 c	4.87 ± 0.05 d	1.24 ± 0.01 b	0.14 ± 0.01 b	1.40 ± 0.01 c

Values are means ± standard deviations except for the relative crystallinity. Letters a to d represent a significant difference between the data in the same column (*p* < 0.05).

**Table 2:** Water distribution of dough samples with different bamboo leaf flavonoid contents.

BLF Amount (g/100 g Wheat Flour)	Vicinal Water	Multilayer Water	Entrapped Water	Free Water
0	0.2133 ± 0.0528 a	0.1797 ± 0.0615 a	0.5703 ± 0.0250 b	0.0125 ± 0.0050 a
0.3	0.1332 ± 0.0194 b	0.1827 ± 0.0137 a	0.6687 ± 0.0119 a	0.0127 ± 0.0054 a
0.5	0.1256 ± 0.0487 b	0.1843 ± 0.0235 a	0.6886 ± 0.0480 a	0.0146 ± 0.0040 a
0.8	0.0867 ± 0.0287 b	0.1847 ± 0.0134 a	0.7173 ± 0.0434 a	0.0155 ± 0.0039 a

Values are means ± standard deviations except for the relative crystallinity. Letters a to b represent a significant difference between the data in the same column (*p* < 0.05).

## Data Availability

The original contributions presented in the study are included in the article, further inquiries can be directed to the corresponding author.
